# Intermedin_1-53_ attenuates atherosclerotic plaque vulnerability by inhibiting CHOP-mediated apoptosis and inflammasome in macrophages

**DOI:** 10.1038/s41419-021-03712-w

**Published:** 2021-05-01

**Authors:** Jin-Ling Ren, Yao Chen, Lin-Shuang Zhang, Ya-Rong Zhang, Shi-Meng Liu, Yan-Rong Yu, Mo-Zhi Jia, Chao-Shu Tang, Yong-Fen Qi, Wei-Wei Lu

**Affiliations:** 1 Key Laboratory of Molecular Cardiovascular Science, Ministry of Education, Peking University Health Science Center, 100191 Beijing, China; 2 Department of Pathogen Biology, School of Basic Medical Sciences, Peking University Health Science Center, 100191 Beijing, China; 3 Department of Physiology, School of Basic Medical Sciences, Chongqing Medical University, 400016 Chongqing, China; 4 Department of Physiology and Neurobiology, Medical College of Soochow University, 215123 Suzhou, China

**Keywords:** Drug discovery, Atherosclerosis

## Abstract

Atherosclerotic plaque vulnerability and rupture increase the risk of acute coronary syndromes. Advanced lesion macrophage apoptosis plays important role in the rupture of atherosclerotic plaque, and endoplasmic reticulum stress (ERS) has been proved to be a key mechanism of macrophage apoptosis. Intermedin (IMD) is a regulator of ERS. Here, we investigated whether IMD enhances atherosclerotic plaque stability by inhibiting ERS-CHOP-mediated apoptosis and subsequent inflammasome in macrophages. We studied the effects of IMD on features of plaque vulnerability in hyperlipemia apolipoprotein E-deficient (ApoE^−/−^) mice. Six-week IMD_1-53_ infusion significantly reduced atherosclerotic lesion size. Of note, IMD_1-53_ lowered lesion macrophage content and necrotic core size and increased fibrous cap thickness and vascular smooth muscle cells (VSMCs) content thus reducing overall plaque vulnerability. Immunohistochemical analysis indicated that IMD_1-53_ administration prevented ERS activation in aortic lesions of ApoE^−/−^ mice, which was further confirmed in oxidized low-density lipoproteins (ox-LDL) induced macrophages. Similar to IMD, taurine (Tau), a non-selective ERS inhibitor significantly reduced atherosclerotic lesion size and plaque vulnerability. Moreover, C/EBP-homologous protein (CHOP), a pro-apoptosis transcription factor involved in ERS, was significantly increased in advanced lesion macrophages, and deficiency of CHOP stabilized atherosclerotic plaques in AopE^−/−^ mice. IMD_1-53_ decreased CHOP level and apoptosis in vivo and in macrophages treated with ox-LDL. In addition, IMD_1-53_ infusion ameliorated NLRP3 inflammasome and subsequent proinflammatory cytokines in vivo and in vitro. IMD may attenuate the progression of atherosclerotic lesions and plaque vulnerability by inhibiting ERS-CHOP-mediated macrophage apoptosis, and subsequent NLRP3 triggered inflammation. The inhibitory effect of IMD on ERS-induced macrophages apoptosis was probably mediated by blocking CHOP activation.

## Facts

- Atherosclerotic plaque vulnerability and rupture are thought to account for the majority of acute coronary syndromes. Advanced lesion macrophage apoptosis plays important roles in the rupture of atherosclerotic plaque.
- ERS has been proved to be a key mechanism of macrophage apoptosis, and CHOP, a branch of the ERS, is considered as an important molecular target for stabilizing atherosclerotic plaque by suppressing macrophage apoptosis.
- IMD, a newfound peptide of the calcitonin/calcitonin gene-related peptide family, has been reported to alleviate many cardiovascular diseases by inhibiting ERS.

## Questions

- What is the effect of intermedin on atherosclerotic plaque vulnerability and the specific mechanism involved?
- What are the mechanisms underlying the regulation of CHOP-mediated macrophage apoptosis in advanced atherosclerotic lesions?
- Will intermedin ameliorate NLRP3 inflammasome and subsequent inflammation by inhibiting macrophage apoptosis?

## Introduction

Atherosclerosis is a chronic inflammatory disease triggered by retention of lipids in large and medium-sized arteries^1–3^. Atherosclerotic plaque vulnerability and rupture are thought to account for the majority of acute coronary syndromes including acute ischemic stroke and myocardial infarction, which are the leading cause of mortality worldwide^4–6^. In humans, rupture-prone plaques have increased plaque lipid content and large necrotic cores covered by thin fibrous caps, both intimal and adventitial inflammation, and increased apoptosis of vascular smooth muscle cells (VSMCs) and macrophages^7,8^. During the development of atherosclerotic plaques, macrophages can dominate both disease initiation and progression via the production and release of various cytokines and proteases^9^. Macrophage apoptosis occurs throughout all stages of atherosclerosis and plays important roles in plaque regression and plaque vulnerability. In late lesions, defective phagocytic clearance of apoptotic macrophages may lead to a proinflammatory response by the inflammasome, accompanied by the generation of the necrotic core^9–11^. Increasing evidence suggests that advanced lesion macrophage apoptosis and inflammation is associated with necrotic core expansion and fibrous cap thinning^12–14^.

Endoplasmic reticulum stress (ERS) and activation of the unfolded protein response (UPR) have been suggested to contribute to the progression of plaque vulnerability and the occurrence of acute complications of coronary atherosclerosis^10^. Many of the stimuli relevant to atherothrombosis induce the UPR as a stress response pathway, which is chronically activated in atherosclerotic lesion cells, particularly in macrophages of advanced lesion^15^. C/EBP-homologous protein (CHOP), or GADD153, is the branch of the UPR through the protein kinase R-like endoplasmic reticulum kinase (PERK) arm that has been associated most with apoptosis^16^. In particular, short-term induction of CHOP is an important element of the protective UPR, whereas prolonged expression of CHOP can lead to cell death^17,18^. Free cholesterol (FC) loading led to UPR activation and CHOP induction in macrophages, while macrophages from CHOP^−/−^ mice showed *~*70% protection from FC-induced apoptosis^19^. Clinical studies showed that robust apoptosis and activation of the ERS pathway, including the induction of CHOP, were present in macrophages within ruptured plaques of humans, but not within stable fibrous plaques^20^. Furthermore, the levels of ERS markers were significantly increased in atherectomy specimens from patients with unstable angina pectoris^20^. While CHOP deficiency in atherosclerotic mice strikingly alleviated macrophages apoptosis and reduced rates of plaque rupture^21^. Therefore, ERS contributes to triggering and orchestrating the atherosclerotic plaque vulnerability, and CHOP could be an important molecular target for stabilizing atherosclerotic plaque by suppressing macrophage apoptosis^10,22^. However, the mechanisms underlying the regulation of CHOP in advanced lesions remain to be elucidated.

Intermedin (IMD), a newfound peptide of the calcitonin/calcitonin gene-related peptide family, is involved in maintaining circulatory homeostasis and alleviating cardiovascular diseases^23,24^. Human IMD gene encodes a prepropeptide of 148 amino acids with a signal peptide for secretion at the N terminus. IMD_1-53_ can be generated from prepro-IMD by proteolytic cleavage at Arg93–Arg94^24^. Previously, we and others have reported that IMD reduced atherosclerotic lesions in ApoE^−/−^ mice by inhibiting the foam-cell formation of macrophages^25^ and modifying lipid profiles^26^. Moreover, IMD inhibition of ERS greatly prevented the development of abdominal aortic aneurysms^27^, ameliorated myocardial impairment induced by ischemia/reperfusion^28^, and suppressed pressure-overload cardiac hypertrophy^29^. However, the effect of IMD on atherosclerotic plaque vulnerability and the specific mechanism involved is still unknown.

In this study, we aimed to explore the role of IMD in promoting atherosclerotic plaque stability and, furthermore, to test the hypothesis that inhibition of ERS-CHOP-mediated macrophage apoptosis and subsequent inflammasome partly contributed to the plaque stabilizing effects of IMD.

## Results

### IMD_1-53_ reduced atherosclerotic lesions and attenuated plaque vulnerability

**Fig. 1:**
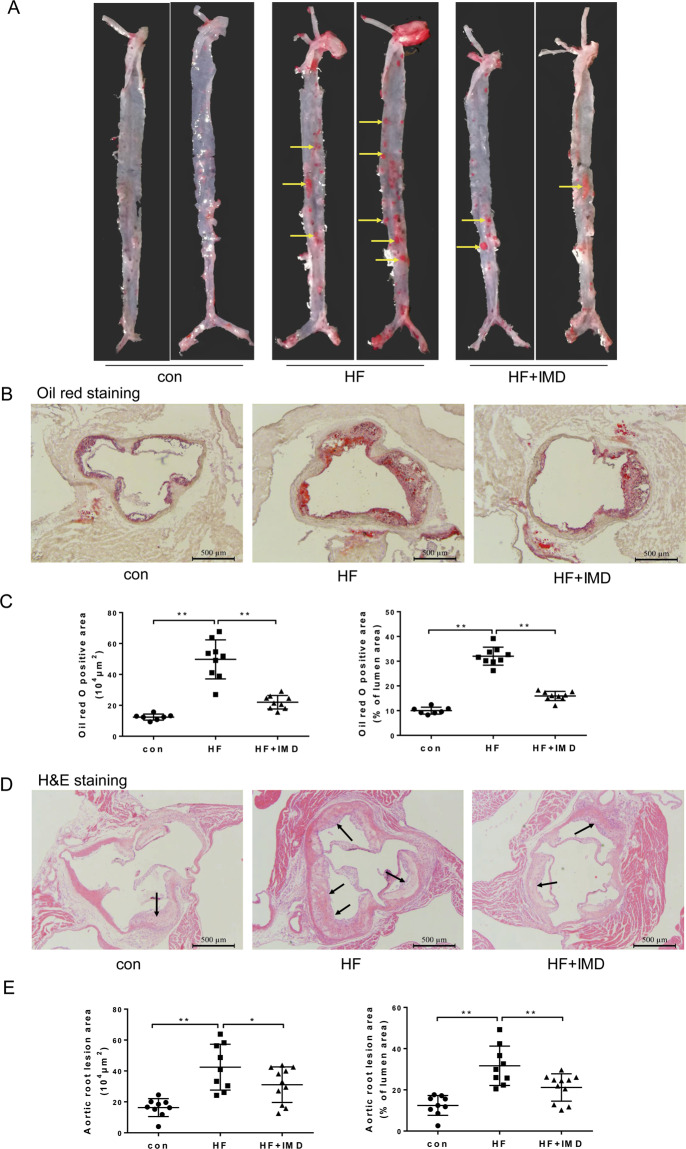
IMD_1-53_ alleviated atherosclerotic lesions at the aortic root of ApoE^−/−^ mice. Eight-week-old male ApoE^−/−^ were fed a standard chow diet (con) or a high-fat diet (HFD) for 16 weeks. After 10 weeks of HFD feeding, ApoE^–/–^ mice received either PBS or intermedin_1-53_ (IMD_1-53_) during the left 6 weeks of high-fat diet feeding. **A** Gross view of en face-prepared aortas stained with Oil Red O. The red dots are lesions stained positively for Oil Red O. Yellow arrows pointed to the Oil red O-stained lesions. **B** Representative image of Oil red O-stained aortic root lesions. Scale bars, 500 μm. **C** Quantification of Oil red O positive area. **D** Representative image of H&E-stained aortic root lesion. Black arrows indicate the atherosclerotic lesions. Scale bars, 500 μm. **E** Quantification of aortic root lesion area. *n* = 7–11. Data are mean ± SD. ^*^P < 0.05, ^**^P < 0.01; one-way ANOVA.

To investigate the role of IMD in atherosclerosis, we first investigated IMD expression in atherosclerotic lesions. Consistent with our previous report, the protein content of IMD in the lesion was significantly decreased by 65%, and the IMD mRNA expression in aortas was significantly decreased by 96% in the HF group, compared with the control group. (Supplementary Fig. 1A, B). We further confirmed the protective role of IMD in atherosclerosis development. IMD_1-53_ did not affect body weight. Consistently, IMD_1-53_ significantly improved the plasma lipid profile with lower TC, TG, and LDL-C levels, as compared to ApoE^−/−^ mice (Supplementary Fig. 1D). Unexpectedly, we found IMD_1-53_ further decreased HDL-C levels as compared with ApoE^−/−^ mice (Supplementary Fig. 1D). En face-prepared aortas stained with Oil Red O revealed a significant reduction in lesion area in IMD-treated ApoE^−/−^ mice (Fig. 1A). In addition, IMD_1-53_ treatment resulted in a 57% and 23% reduction in lipid content (Fig. 1B, C) and atherosclerotic lesion area (Fig. 1D, E), respectively, in the aortic root.

**Fig. 2:**
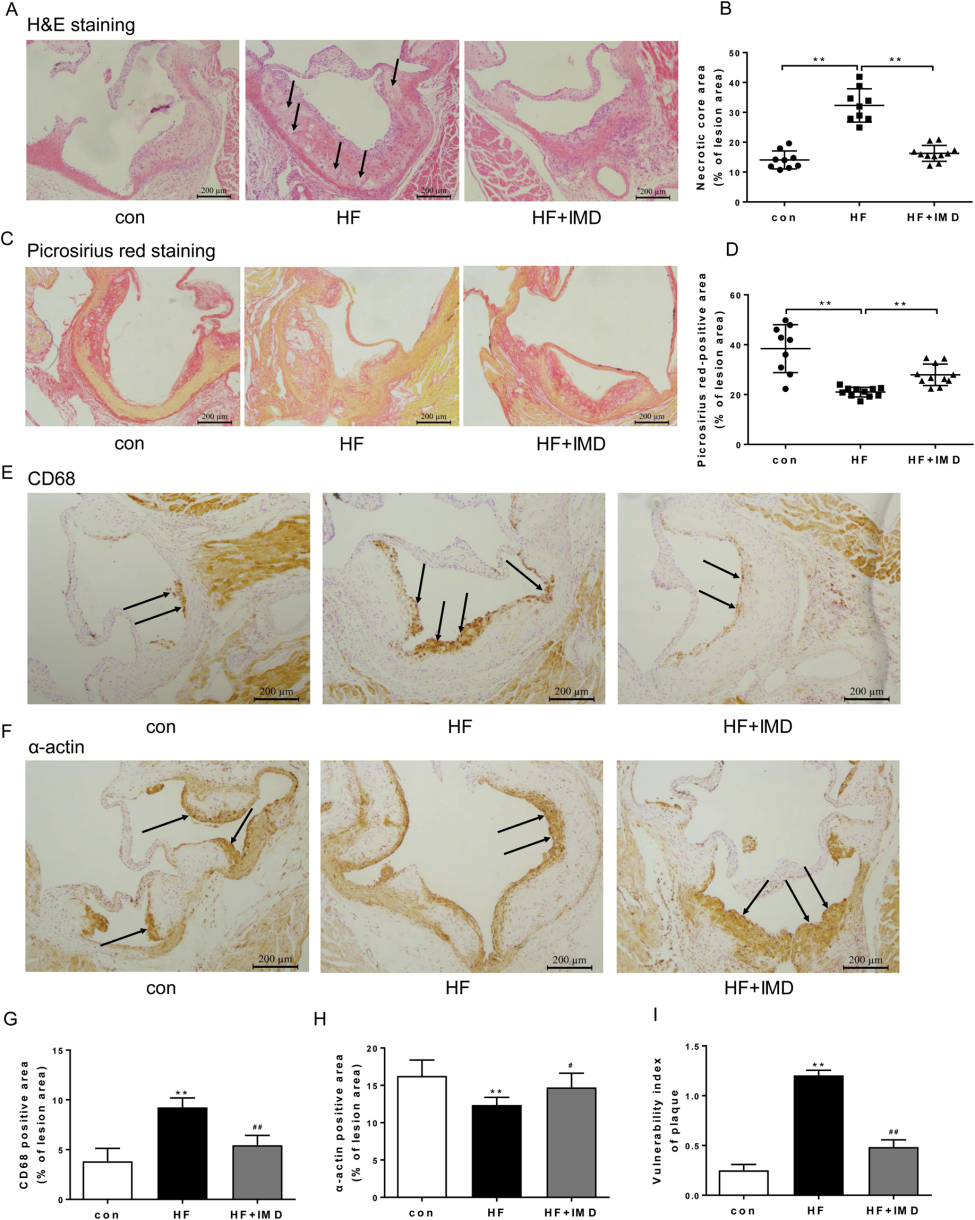
IMD_1-53_ promoted atherosclerotic plaque stability at the aortic root of ApoE^−/−^ mice. Eight-week-old male ApoE^−/−^ were fed a standard chow diet (con) or a high-fat diet (HFD) for 16 weeks. After 10 weeks of HFD feeding, ApoE^−/−^ mice received either PBS or intermedin_1-53_ (IMD_1-53_) during the left 6 weeks of high-fat diet feeding. **A**, **B** Necrotic core area at the aortic root was measured. Representative H&E-stained sections (**A**) from each group are displayed next to the quantification data (**B**). Black arrows indicate the necrotic core. Scale bars, 200 μm. **C**, **D** Collagen area at the aortic root was measured. Representative picrosirius red-stained sections (**C**) from each group are displayed next to the quantification data (**D**). Scale bars, 200 μm. *n* = 9–11. Data are mean ± SD. ^*^P < 0.05, ^**^P < 0.01^;^ one-way ANOVA. **E**–**H** Representative images of CD68 (**E**) and α-SMA (**F**) immunohistochemical staining and quantitative analysis of macrophage (**G**) and vascular smooth muscle cell (VSMC) (**H**) contents at the aortic root of mice from each group. Black arrows indicate cells stained positively for CD68 or α-SMA. Scale bars, 200 μm. **I** Quantitative analysis of plaque vulnerability index at the aortic root of mice from each group. *n* = 6. Data are mean ± SD. ^**^P < 0.01 compared with con, ^#^*P* < 0.05, ^##^*P* < 0.01 compared with HF group; one-way ANOVA.

Next, to determine the effect of IMD on features of plaque vulnerability, we focused on atherosclerotic advanced lesions. The phenotypic characteristics of vulnerable plaques include increased lipid-rich necrotic core size and macrophage contents, decreased thickness of fibrous cap, plaque collagen, and SMC contents, all of which have been used as indicators of plaque vulnerability. Analysis of necrotic cores showed that IMD significantly reduced the size of the necrotic cores in the atherosclerotic lesions as compared with ApoE^−/−^ mice of the HFD group (Fig. 2A, B). Picrosirius red staining showed that IMD decreased collagen contents in the atherosclerotic lesions of ApoE^−/−^ mice (Fig. 2C, D). Further, immunostaining for macrophage and SMC markers showed that macrophage contents were increased but the SMC contents were decreased in the lesions of IMD-treated ApoE^−/−^ mice as compared with ApoE^−/−^ mice of the HFD group (Fig. 2E–H). Based on these quantification data, the histological plaque vulnerability index was then calculated and confirmed that IMD treatment led to significantly attenuated plaque vulnerability in ApoE^−/−^ mice fed with HFD (Fig. 2I). Collectively, these results demonstrated that IMD decreased atherosclerotic lesion size and plaque vulnerability in ApoE^−/−^ mice fed with HFD.

### IMD_1-53_ promoted atherosclerotic plaque stability by inhibiting ERS

**Fig. 3:**
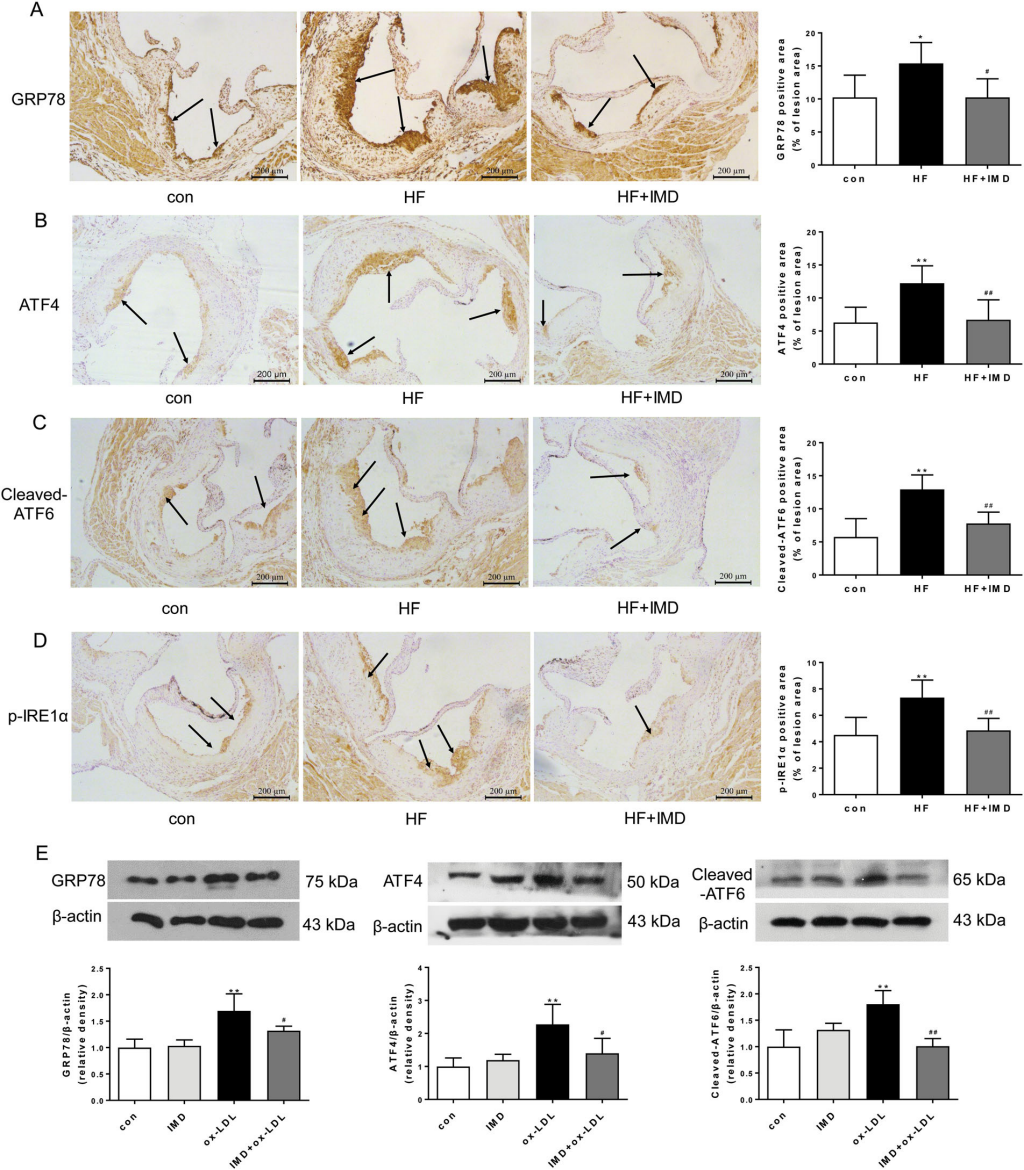
IMD_1-53_ inhibited endoplasmic reticulum stress (ERS) in atherosclerotic lesions. **A**–**D** Eight-week-old male ApoE^−/−^ were fed a standard chow diet (con) or a high-fat diet (HFD) for 16 weeks. After 10 weeks of HFD feeding, ApoE^–/–^ mice received either PBS or intermedin_1-53_ (IMD_1-53_) during the left 6 weeks of high-fat diet feeding. Representative images and quantification data of GTP78 (**A**), ATF4 (**B**), cleaved ATF6 (**C**), and p-IRE1α (**D**) immunohistochemical staining at the aortic root of mice from each group. Black arrows indicate the area stained positively for GTP78, ATF4, cleaved ATF6, or p-IRE1α. Scale bars, 200 μm. *n* = 6. Data are mean ± SD. ^*^P < 0.05, ^**^P < 0.01 compared with con, ^#^*P* < 0.05, ^##^*P* < 0.01 compared with HF group; one-way ANOVA. **E** Western blot analysis of protein expression of GRP78, ATF6, and ATF4 in macrophages treated with PBS, IMD_1-53_, ox-LDL, and IMD_1-53_ + ox-LDL. β-actin was a control for protein loading. Results are representative of four experiments. Densitometric analysis of protein levels is shown as a ratio to β-actin. *n* = 4. Data are mean ± SD. ^**^P < 0.01 compared with con, ^#^*P* < 0.05, ^##^*P* < 0.01 compared with the ox-LDL group; one-way ANOVA.

Multiple lines of evidence have revealed that ERS contributed to the progression of atherosclerotic plaques, pathogenesis of plaque vulnerability, and subsequent plaque rupture. To further determine the mechanisms underlying IMD-promoted plaque stabilization, we investigated whether IMD affected ERS in aortic root lesions of ApoE^−/−^ mice. As expected, immunostaining of ERS markers showed that GRP78, ATF4, cleaved ATF6, and p-IRE1α were significantly increased in the HFD group, compared with the control group (Fig. 3A–D). In addition, ERS marker ATF4 is specially increased in macrophages of the lesions as shown by co-staining of ATF4 with macrophage marker CD68 (Supplementary Fig. 2A). While IMD_1-53_ treatment reduced the overexpression of GRP78, ATF4, cleaved ATF6, and p-IRE1α proteins in HFD- ApoE^−/−^ mice by 34%, 45%, 40%, 34%, respectively (Fig. 3A–D). In vitro, ox-LDL increased ERS markers in time- and concentration-dependent manner (Supplementary Fig. 2B). Western blot analysis of GRP78, ATF4, and cleaved ATF6 further confirmed the inhibition of ox-LDL-induced ERS in Raw 264.7 cells by IMD_1-53_ treatment (Fig. 3E).

To determine whether IMD attenuated plaque vulnerability by inhibiting ERS, we used non-selective ERS inhibitor Tau, a chemical chaperone involved in stabilizing protein conformation, improving folding capacity, and inhibiting ERS, as a positive control. Similar to IMD, Tau significantly reduced lipid content and lesion area according to the quantification based on Oil Red O staining and H&E staining in the aortic root, as well as en face analysis (Supplementary Fig. 3). Moreover, Tau also significantly alleviated plaque vulnerability as shown by quantification of the size of necrotic cores, and collagen, VSMC, and macrophage contents (Supplementary Fig. 4). These results suggested that IMD could stabilize atherosclerotic plaque partly by inhibition of ERS.

### CHOP deficiency stabilized atherosclerotic plaque

**Fig. 4:**
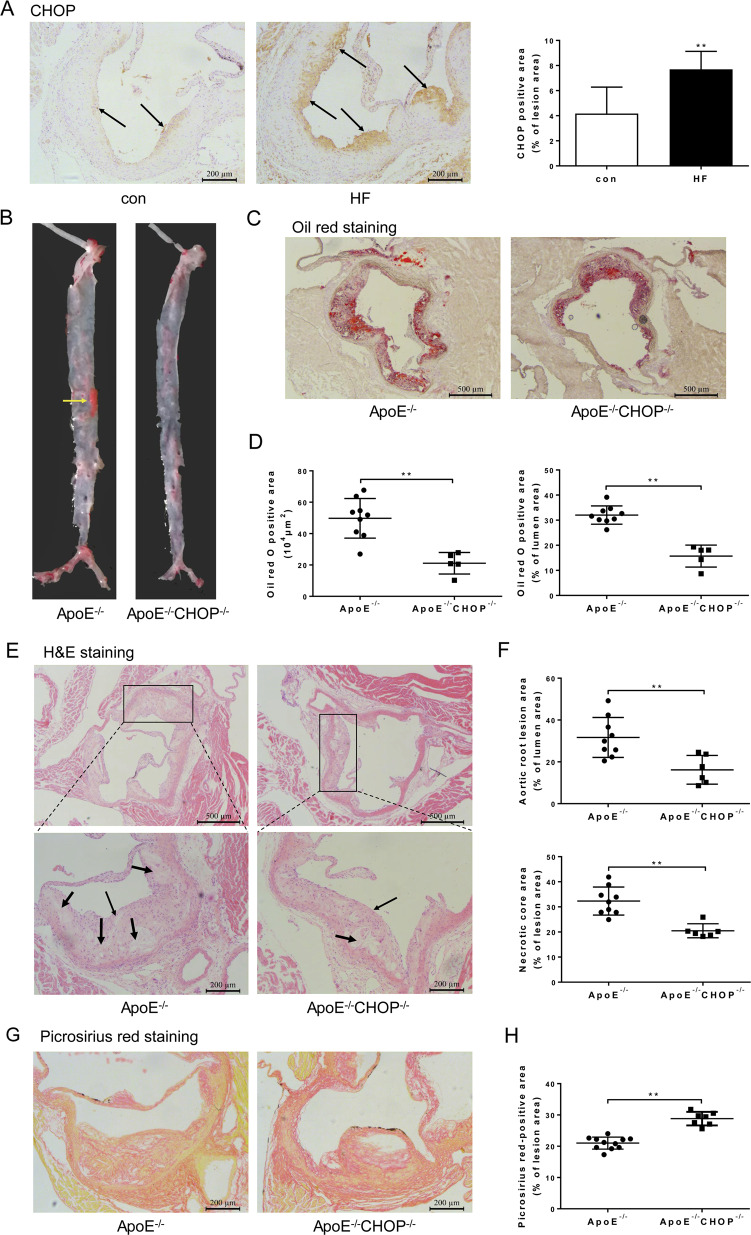
CHOP deficiency ameliorated atherosclerotic plaque vulnerability at aortic root of ApoE^−/−^ mice. **A** Representative images and quantification data of CHOP immunohistochemical staining at the aortic root. Black arrows indicate the area stained positively for CHOP. Scale bars, 200 μm. *n* = 6. **B**–**G** Eight-week-old male ApoE^−/−^CHOP^−/−^ mice and ApoE^−/−^ littermates were fed a high-fat diet for 16 weeks. **B** Gross view of en face-prepared aortas stained with Oil Red O. The red dots are lesions stained positively for Oil Red O. Yellow arrows pointed to the Oil red O-stained lesions. **C** Representative image of Oil red O-stained aortic root lesion. Scale bars, 500 μm. **D** Quantification of Oil red O positive area. **E** Representative image of H&E-stained aortic root lesion. Thin black arrows indicate the atherosclerotic lesions. Thick black arrows indicate the necrotic core. Scale bars, 500 μm (top) and 200 μm (bottom). **F** Quantification of aortic root lesion area (top) and necrotic area (bottom). **G**, **H** Collagen area at the aortic root was measured. Representative picrosirius red-stained sections (**G**) from each group are displayed next to the quantification data (**H**). Scale bars, 200 μm. *n* = 5–11. Data are mean ± SD. ^*^P < 0.05, ^**^P < 0.01; Student’s *t* test.

Increasing evidence suggests that advanced lesion macrophage apoptosis is associated with atherosclerotic plaque vulnerability. The proapoptotic ATF4/CHOP signaling pathway, one branch of UPR, has been proved to be involved in the ERS-induced macrophage apoptosis in advanced lesions. To further explore the mechanisms underlying the stabilizing effect of IMD on the atherosclerotic plaque, we first checked the expression of CHOP in the lesion and then used CHOP-deficient atherosclerosis mice (ApoE^−/−^CHOP^−/−^) (Supplementary Fig. 5A) to confirm the role of CHOP-mediated macrophage apoptosis in plaque vulnerability. Immunostaining of CHOP demonstrated that CHOP was markedly induced in the aortic root lesions of high-fat-treated ApoE^−/−^ mice (Fig. 4A). Furthermore, as shown in Supplementary Fig. 5, CHOP and apoptosis marker cleaved caspase-3 were specially expressed in macrophages of the lesions of high-fat-treated ApoE^−/−^ mice as shown by co-staining of CHOP and cleaved caspase-3 with macrophage marker CD68 (Supplementary Fig. 5B, C). As expected, CHOP deficiency significantly decreased necrotic core area (Fig. 4B), atherosclerotic lesion area (Fig. 4E, F), and lipid content (Fig. 4C, D) in the aortic root. In addition, Picrosirius red staining showed that deficiency of CHOP increased collagen content in the atherosclerotic lesions of the aortic root (Fig. 4G, H). Thus, these data supported at least a partial role for CHOP-induced apoptosis in the death of advanced lesion macrophages, which indicated CHOP as a potential target to treat atherosclerotic plaque vulnerability.

### IMD_1-53_ attenuated CHOP level and apoptosis of macrophages in advanced atherosclerotic lesions

**Fig. 5:**
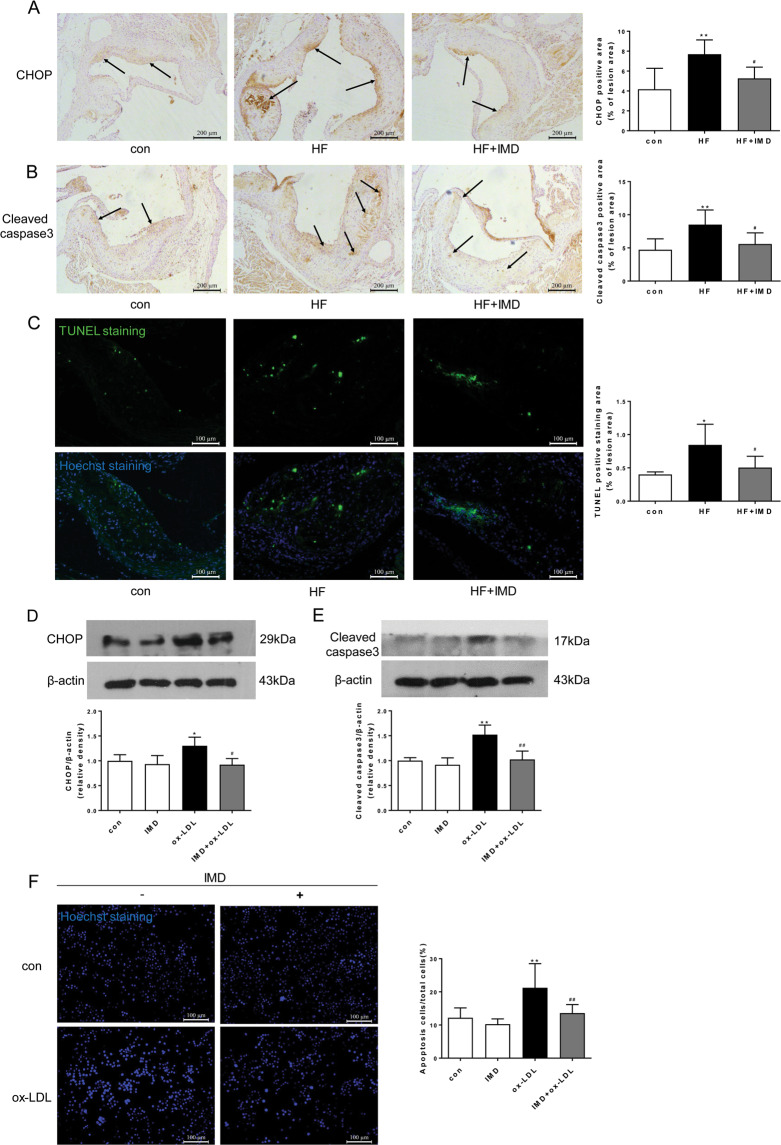
IMD_1-53_ decreased the CHOP level and apoptosis in vivo and in vitro. **A**–**C** Eight-week-old male ApoE^−/−^ were fed a standard chow diet (con) or a high-fat diet (HFD) for 16 weeks. After 10 weeks of HFD feeding, ApoE^–/–^ mice received either PBS or intermedin_1-53_ (IMD_1-53_) during the left 6 weeks of high-fat diet feeding. Representative images and quantification data of CHOP (**A**) and cleaved caspase-3 (**B**) immunohistochemical staining, and terminal deoxynucleotidyl transferase (TdT) dUTP nick-end labeling (TUNEL) staining (**C**) at the aortic root of mice from con, HF, and HF + IMD. Black arrows indicate the area stained positively for CHOP or cleaved caspase-3. Scale bars, 200 μm (**A**, **B**) and 100 μm (**C**). *n* = 6. Data are mean ± SD. ^*^P < 0.05, ^**^P < 0.01 compared with Con, ^#^*P* < 0.05 compared with HF group; one-way ANOVA. **D**, **E** Western blot analysis of protein expression of CHOP (**D**) and cleaved-caspase3 (**E**) in macrophages treated with PBS, IMD_1-53_, ox-LDL, and IMD_1-53_ + ox-LDL. β-actin was a control for protein loading. Results are representative of four experiments. Densitometric analysis of protein levels is shown as a ratio to β-actin. *n* = 4. Data are mean ± SD. ^*^P < 0.05, ^**^P < 0.01 compared with con, ^#^*P* < 0.05, ^##^*P* < 0.01 compared with the ox-LDL group; one-way ANOVA. **F** Representative images and quantification data of Hoechst staining in macrophages treated with PBS, IMD_1-53_, ox-LDL and IMD_1-53_ + ox-LDL. Scale bars, 100 μm. *n* = 6. Data are mean ± SD. ^**^P < 0.01 compared with con, ^##^*P* < 0.01 compared with ox-LDL group; one-way ANOVA.

We further investigated whether IMD attenuated plaque vulnerability by inhibiting CHOP-mediated macrophage apoptosis. We first analyzed the expression of CHOP and apoptosis marker cleaved caspase-3 in the advanced lesion of the aortic root. As shown by immunostaining, IMD_1-53_ treatment significantly decreased the induction of CHOP and cleaved caspase-3 in the aortic root lesions of ApoE^−/−^ mice (Fig. 5A*,* B). In addition, TUNEL staining confirmed that IMD markedly suppressed the apoptosis in the vulnerable plaque of ApoE^−/−^ mice (Fig. 5C). In vitro, we treated macrophages with ox-LDL, and found ox-LDL significantly induced CHOP expression in time- and concentration-dependent manner (Supplementary Fig. 5D) and cleaved caspase-3 as well (Fig. 5E), whereas IMD remarkably inhibited their induction (Fig. 5D, E). Furthermore, the Hoechst staining also confirmed the anti-apoptotic effect of IMD on macrophages induced by ox-LDL (Fig. 5F). Therefore, IMD may attenuate macrophage apoptosis and destabilization of atherosclerotic lesions partly by inhibition of ERS-induced CHOP expression.

### IMD_1-53_ inhibited inflammasome in macrophages of advanced atherosclerotic lesions

**Fig. 6:**
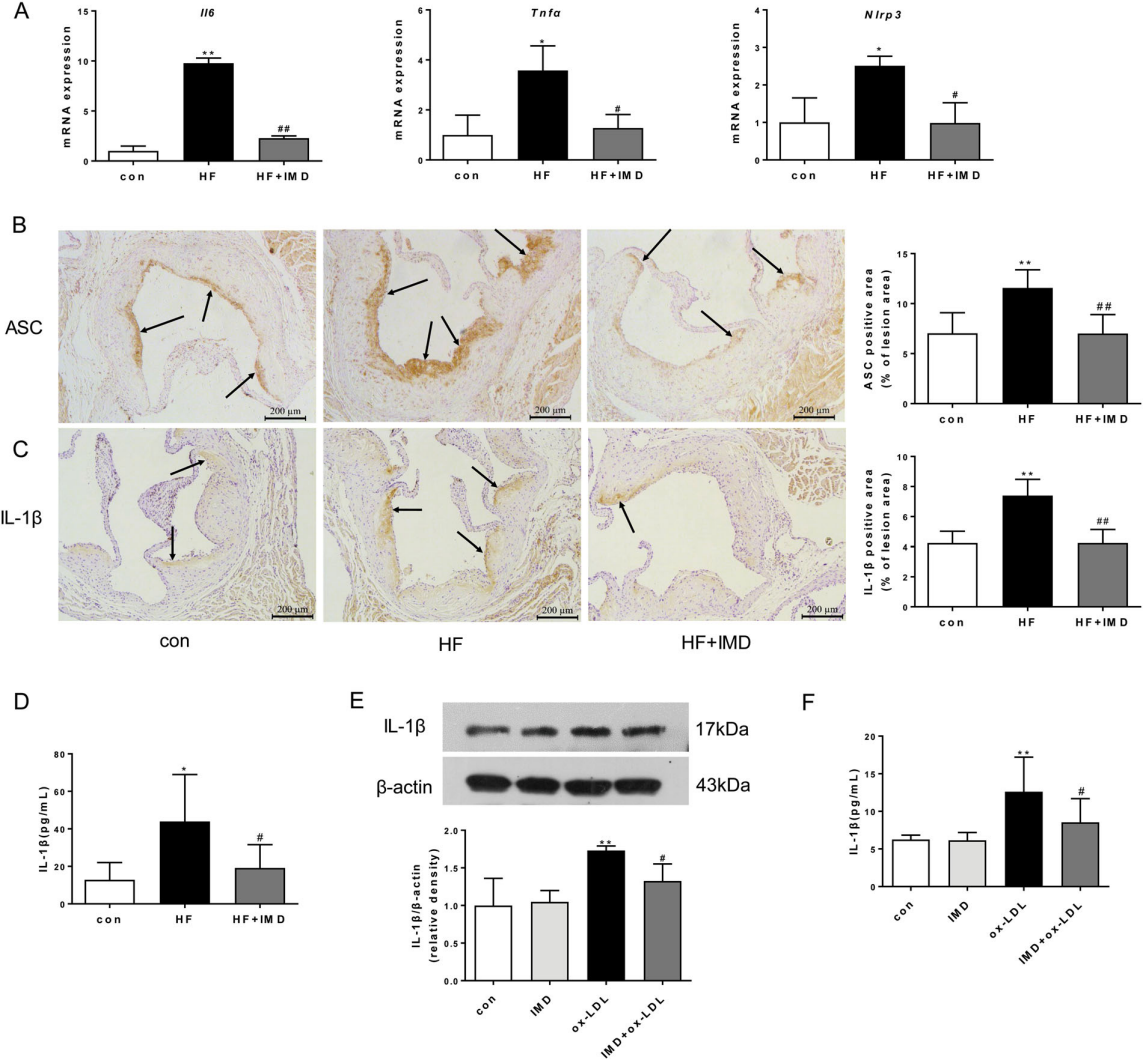
IMD_1-53_ inhibited apoptosis-triggered inflammasome in advanced lesional macrophage. **A**–**C** Eight-week-old male ApoE^−/−^ were fed a standard chow diet (con) or a high-fat diet (HFD) for 16 weeks. After 10 weeks of HFD feeding, ApoE^–/–^ mice received either PBS or intermedin_1-53_ (IMD_1-53_) during the left 6 weeks of high-fat diet feeding. Quantitative real-time PCR of interleukin6 (*IL6*), tumor necrosis factor α (*Tnfα*), and *Nlrp3* mRNA expression (**A**). Results are relative to the GAPDH level (*n* = 3). Representative images and quantification data of apoptosis-associated speck-like protein containing CARD (ASC) (**B**) and IL-1β (**C**) immunohistochemical staining at the aortic root of mice from con, HF, and HF + IMD. Black arrows indicate the area stained positively for ASC or IL-1β. Scale bars, 200 μm. *n* = 6. **D** Plasma IL-1β level was measured. *n* = 6. Data are mean ± SD. ^*^P < 0.05 and ^**^P < 0.01 compared with con, ^#^*P* < 0.05 and ^##^*P* < 0^.^01 compared with HF group; one-way ANOVA. **E** Western blot analysis of protein expression of IL-1β in macrophages treated with PBS, IMD_1-53_, ox-LDL and IMD_1-53_ + ox-LDL. β-actin was a control for protein loading. Results are representative of four experiments. Densitometric analysis of protein levels is shown as a ratio to β-actin. *n* = 4. **F** IL-1β in the cell culture supernatant was also measured. *n* = 5. Data are mean ± SD. ^**^P < 0.01 compared with con, ^#^*P* < 0.05 compared with the ox-LDL group; one-way ANOVA.

Accumulation of dead cells may release nuclear double-stranded (ds)DNA, which could be identified by inflammasome leading to the release of interleukin-1β (IL-1β) and IL-18. NLRP3 inflammasome-triggered inflammatory cascade has been connected to atherosclerosis, while inhibition of inflammasome enhanced atherosclerotic lesion stability. To study whether inhibition of CHOP-mediated macrophage apoptosis by IMD could ameliorate the inflammasome-mediated inflammation, we checked the NLRP3 inflammasome and proinflammatory cytokines in the aortas. In parallel with the striking reduction of proinflammatory cytokines (*Il6* and *Tnfα*) and *Nlrp3* mRNA expression in the aortas of IMD-treated ApoE^−/−^ mice (Fig. 6A), we found IMD_1-53_ significantly reduced ASC and IL-1β expression in the atherosclerotic lesions as shown by immunohistochemical staining (Fig. 6B, C). In addition, plasma IL-1β was markedly reduced by IMD_1-53_ in ApoE^−/−^ mice (Fig. 6D). In line with the in vivo results, IMD_1-53_ also strikingly decreased IL-1β expression and secretion stimulated by ox-LDL in macrophages (Fig. 6E, F). Taken together, these results were in agreement with inhibition of CHOP and an overall improvement of histopathologic features of lesion stability in IMD-treated ApoE^−/−^ mice.

## Discussion

Vulnerable atherosclerotic plaques increase the risk of acute coronary syndromes that are caused by the sudden rupture of the plaques and the subsequent life-threatening coronary thrombosis^5,6^. Fatal plaque rupture sites show large necrotic cores combined with high levels of inflammation and thin layers of collagen^30^. Macrophage apoptosis in advanced lesions plays important roles in the rupture of atherosclerotic plaque^13^, and ERS has been proved to be a key mechanism of macrophage apoptosis^9,31^. Recent studies from our and other groups have revealed that IMD exerts anti-ERS^27,29,32–34^ and anti-apoptotic^27,29,35,36^ effects in many ERS-injured diseases, suggesting a possible protective role of IMD on atherosclerotic plaque vulnerability. In this study, we reported for the first time that treating ApoE^−/−^ mice with IMD could alleviate atherosclerotic plaque vulnerability and the inflammasome-triggered inflammatory cascade by inhibiting macrophage apoptosis via suppressing the activation of the ERS-CHOP pathway.

IMD, a novel peptide of the CGRP family, is widely expressed in the body and acts non-selectively on both CGRP and adrenomedullin receptors, which are richly expressed in macrophages^23^. Administration of IMD in vivo protects the cardiovascular system against ERS^27,29,32–34^, apoptosis^27,29,35,36^, oxidative damage^28,35,37^, and inflammation^34,38,39^ in various animal disease models, such as atherosclerosis^25,26,40^, ischemia-reperfusion injury^28,41,42^, vascular calcification^32,43^, and cardiac hypertrophy^29,44^, so it is an endogenous cardiovascular-protective peptide. Previous studies have reported that IMD attenuated atherosclerosis in ApoE^−/−^ mice by modifying lipid profiles^40^ and inhibiting foam-cell formation of macrophages^25,26^. Here, we described a novel effect of IMD in atherosclerotic plaque vulnerability in ApoE^−/−^ mice. Consistent with previous studies, our present study demonstrated that IMD significantly decreased in advanced lesions and exogenous supplementation of IMD markedly reduced the atherosclerotic lesion size. Of note, IMD greatly stabilized atherosclerotic plaque, which suggested that this peptide acted as a regulatory autocrine or paracrine modulator of plaque stability and may extend the potential therapeutic use of IMD for advanced atherosclerosis.

The potential mechanisms for the protective effects of IMD in plaque vulnerability were not well explained. A number of cellular events contribute to vulnerable plaque formation, including secretion of proinflammatory, proteolytic molecules by macrophages as well as apoptosis of macrophages^45^. The necrotic core, arising from a combination of macrophage apoptosis and efferocytosis in the lesions, is a key factor in plaque vulnerability^13,45^. Activation of ERS is associated with the severity and clinical complications of atherosclerosis in humans^15^. Of note, directional coronary atherectomy specimens demonstrated that ruptured plaques exhibited a markedly increased expression of the ER chaperones GRP78 and CHOP^10^. Moreover, ERS may contribute to necrotic core expansion, and overloaded and sustained ERS is related to macrophage apoptosis in the vulnerable plaques^9^. Therapeutic interventions reducing ERS may be considered promising strategies to reduce atherosclerotic plaque instability. And the inhibitory effect of IMD on ERS activation was well-studied by extensive experiments^27,29,32,33^. In our present study, we observed that the unstable plaques in ApoE^−/−^ mice of the HFD group exhibited higher levels of ERS as reflected by the increased expression of GPR78, ATF4, cleaved caspase-ATF6 and p-IRE1α, which was reversed by IMD treatment. Our previous work demonstrated that IMD mitigated homocysteine-promoted atherosclerotic calcification^32^, and protected against intimal hyperplasia^33^, abdominal aortic aneurysm^27^, and myocardial injury^29^ by blocking the activation of ERS. To further confirm the role of ERS in the protective effect of IMD on plaque vulnerability, we performed an inhibition test with Tau, a non-selective inhibitor of ERS and widely used in ERS function study, as a positive control. Similar to IMD, Tau could reduce the atherosclerotic lesion size and plaque destabilization. Therefore, IMD may alleviate atherosclerotic lesion size as well as plaque vulnerability at least partly by inhibiting ERS.

Accumulated evidence showed that robust CHOP and apoptosis were present in advanced atherosclerotic vulnerable plaques^9,10,21^. While Thorp et al.^46^ observed a decrease of macrophage apoptosis in advanced lesions and plaque necrosis in ApoE^−/−^ mice that are also deficient in CHOP. Furthermore, clinical studies showed a striking correlation among ERS markers, including CHOP, plaque vulnerability, and lesion apoptosis in samples of human coronary artery plaques^20^. By using an atherosclerotic plaque rupture model and CHOP-deficient mice, Tsukano et al.^21^ reported CHOP deficiency in ApoE^–/−^ mice strikingly alleviated macrophages apoptosis and reduced rates of plaque rupture. Our present study provided further analysis of lesion composition and demonstrated that ApoE^−/−^CHOP^−/−^ mice had markedly decreased necrotic core area and a significant increase in lesion collagen, features of stable plaques. Collectively, these results demonstrated that deficiency of CHOP stabilized atherosclerotic plaques in ApoE^−/−^ mice.

We previously reported that IMD could reduce CHOP-mediated apoptosis in the hearts of pressure-overload-induced cardiac hypertrophy^29^ and in aneurysms^27^. Thus, we further determined the effect of IMD on ERS-CHOP-mediated macrophage apoptosis in vivo and in vitro. As expected, we found that IMD treatment restrained CHOP activation and macrophage apoptosis in atherosclerotic lesions. Consistently, IMD also suppressed the ERS-CHOP pathway in macrophages induced by ox-LDL. Mechanistically, AMPKα1 mediates CHOP ubiquitination and proteasomal degradation in macrophages^47^. Our previous study reported that IMD could inhibit CHOP expression via activating AMPK in VSMCs of aneurysms^27,29^. Thus, activation of AMPK may be involved in the inhibition of CHOP by IMD in macrophages. Taken together, these findings indicated that the inhibitory effect of IMD on ERS-CHOP-mediated apoptosis in macrophages might contribute to the stabilizing effect of IMD on atherosclerotic plaques in ApoE^−/−^ mice. Further experiments are needed to identify the direct target genes by which IMD regulates CHOP activation in atherosclerosis.

In recent years, cytosolic pattern recognition receptors like the NLRP3 inflammasome have been connected to atherosclerosis^48,49^. Accumulation of apoptotic cells may release nuclear ds DNA, which could be identified by inflammasome leading to the release of IL-1β and IL-18^49,50^. Inhibition of inflammasome has been reported to enhance atherosclerotic lesion stability^50–52^, so we further confirmed whether inhibition of CHOP-mediated macrophage apoptosis by IMD could also ameliorate the inflammasome-mediated inflammation. In our expectation, IMD markedly reduced NLRP3 and subsequent inflammation in atherosclerotic lesions, as well as macrophages induced by ox-LDL in vitro. As such, our current findings indicated that the inhibitory effect of IMD on ERS-CHOP signaling could block inflammasome to reduce inflammation in vulnerable plaques.

In conclusion, the results of the present study provided a novel insight into the protective effects of IMD against atherosclerotic plaque vulnerability. IMD may attenuate the progression of atherosclerotic lesions and plaque vulnerability by regulating ERS-related macrophage apoptosis, and subsequent NLRP3 triggered inflammation. The inhibitory effect of IMD on ERS and macrophages apoptosis was probably mediated by blocking CHOP activation. The exact molecular mechanism by which IMD inhibited CHOP needs further research. Nevertheless, our findings bring new insights into the potential of IMD for stabilizing atherosclerotic plaques and the CHOP-mediated apoptosis pathway may be a key therapeutic target related to atherosclerotic vulnerability.

## Materials and methods

### Materials

Synthetic human IMD_1-53_ was from Phoenix Pharmaceuticals (Burlingame, CA, USA). Alzet Mini-osmotic Pumps (model 2006) were from DURECT Corp (Cupertino, CA, USA). Primary antibodies for IMD (sc-86272), β-actin (sc-47778), IL-1β (sc-7884), and all horseradish peroxidase (HRP)-conjugated secondary antibodies were from Santa Cruz Biotechnology (Santa Cruz, CA, USA). Primary antibodies for CD68 (ab125212), α-actin (ab5694), glucose-regulated protein78 (GRP78, ab21865), activating transcription factor (ATF4, ab216839), cleaved ATF6 (ab203119), p-inositol-requiring kinase 1 alpha (p-IRE1α, ab48187), CHOP (ab10444), cleaved-caspase-3 (ab13847) were from Abcam PLC (Cambridge, UK). Apoptosis-associated speck-like protein containing CARD (ASC, SAB4501315) were from Sigma-Aldrich (St. Louis, MO, USA). Dylight-labeled secondary antibodies were from EarthOx Life Sciences (Millbrae, CA, USA). The kit for reverse transcription of RNA and SuperReal PreMix Plus for real-time PCR (TIANGEN Biotech, Beijing, China). The oil red O and taurine (Tau) were from Sigma-Aldrich (St. Louis, MO, USA). Oxidized human LDL (ox-LDL, YB-002) was from Yiyuan Biotechnology (Guangzhou, China). Other chemicals and reagents were of analytical grade.

### Animals and treatment

Eight-week-old male ApoE^−/−^ mice were provided by the Animal Center of Peking University Health Science Center (Beijing). All animals were randomly divided into four groups (*n* = 12 per group): (1) control group: mice were fed with normal diet for 16 weeks; (2) high-fat diet group (HF group): mice were fed a high-fat diet for 16 weeks; (3) high-fat diet plus IMD group (HF + IMD group): mice were fed a high-fat diet for totally 16 weeks, and IMD_1-53_ (dissolved in sterile saline) was subcutaneously administered (300 ng/kg/h) to mice after 10-week high-fat diet via Alzert mini-osmotic pumps (model 2006, Cupertino, CA, USA) for 6 weeks; (4) high-fat diet plus taurine group (HF + Tau group): mice were fed a high-fat diet for 16 weeks, and 2% (w/v)Tau was added into drinking water for 6 weeks at the same time as the IMD treatment described above.

Mice lacking the *Chop* gene (C57BL/6 background) were generated as described previously^21,53^ and were provided by professor Jie Du from Beijing institute of heart, lung, and blood vessel diseases, Beijing Anzhen Hospital, Capital Medical University. ApoE^−/−^CHOP^−/−^ mice were generated by crossbreeding ApoE^−/−^ mice (C57BL/6 background) with CHOP^−/−^ mice. All animal care and experimental protocols complied with the Guide for the Care and Use of Laboratory Animals published by the US National Institutes of Health (NIH Publication, 8th Edition, 2011) and were approved by the Animal Care Committee of Peking University Health Science Center (Beijing).

### Cell culture

Raw 264.7 cells were from ATCC and cultured in DMEM (Gibco,12800-017) with 10% FBS under 95% air and 5% CO_2_ at 37 °C. For experiments, Raw 264.7 cells were cultured in a six-well cell culture plate. When reached 70% fusion, cells were divided into four groups: (1) control group: Raw 264.7 cells were normally cultured without treatment; (2) IMD group: Raw 264.7 cells were normally cultured and treated with 10^−7^ mol/L IMD_1-53_ for 24 h; (3) ox-LDL group: Raw 264.7 cells were normally cultured and treated with 100 μg/mL ox-LDL for 24 h; (4) IMD + ox-LDL group: Raw 264.7 cells were normally cultured and pretreated with 10^−7^ mol/L IMD_1-53_ for 1 h and then treated with 100 μg/mL ox-LDL for 24 h. Total protein from cells was prepared for western blot.

### Lipid assay

At the terminal point of the experiment in vivo, blood samples were collected and plasma was obtained by centrifugation at 3000 rpm for 15 min at 4 °C. Plasma lipid levels including total cholesterol (TC), triglycerides (TG), low-density lipoprotein cholesterol (LDL-C), and high-density lipoprotein cholesterol (HDL-C) were measured by using kits from Zhong Sheng Biotechnology (Beijing, China).

### Western blot analysis

Cells lysates were prepared by using lysis buffer and total protein was obtained by centrifugation at 10,000 rpm for 10 min at 4 °C. Equal amounts of total protein were loaded for SDS-PAGE and were then transferred to nitrocellulose membranes. Blot was blocked in 5% nonfat milk for 1 h, and then incubated with primary antibodies for β-actin (1:2000 dilution), GRP78 (1:4000 dilution), cleaved ATF6 (1:500 dilution), ATF4 (1:500 dilution), CHOP (1:500 dilution), cleaved caspase-3 (1:1000 dilution), and IL-1β (1:500) overnight at 4 °C, following horseradish peroxidase-conjugated secondary antibody for 1 h at room temperature. ECL (Applygen Technologies, Beijing, China) was used to visualize protein bands and protein levels were analyzed by using NIH ImageJ software and normalized to β-actin.

### Quantitative real-time PCR analysis

The total RNA from aorta tissue was isolated with Trizol reagent. An amount of 1.0 μg of RNA was used for cDNA synthesis and was then reverse-transcribed by using the kit for reverse transcription of RNA (TIANGEN Biotech, Beijing, China). Real-time PCR amplification involved a 7500 Fast Quantitative PCR System (Applied Biosystems, Inc, USA) and the kit of SuperReal PreMix Plus for real-time PCR (TIANGEN Biotech, Beijing, China). The amount of PCR product formed in each cycle was evaluated by Eva Green fluorescence. The cycle threshold (Ct) was determined as the number of PCR cycles required for a given reaction to reach an arbitrary fluorescence value within the linear amplification range. Relative quantification was performed according to the 2^−^^ΔΔCt^ method, with β-actin serving as a reference. The primers for real-time PCR are in Table 1.

**Table 1:** Primer sequences for quantitative real-time PCR.

Target	Sequence		Annealing temperature (°C)
*Adm2*	Sense	5’-TGCATCAGCCTCCTCTACCT-3’	59
	Antisense	5’-GCTGCAGGTTACTGGAAGGA-3’	
*Il6*	Sense	5’-TAGTCCTTCCTACCCCAATTTCC-3’	60
	Antisense	5’-TTGGTCCTTAGCCACTCCTTC-3’	
*Tnfα*	Sense	5’-CCCTCACACTCAGATCATCTTCT-3’	60
	Antisense	5’-GCTACGACGTGGGCTACAG-3’	
*Nlrp3*	Sense	5’-CCTTTGACGAGCACATTGG-3’	60
	Antisense	5’-CTTGGGCAGCAGTTTCTTTC-3’	
*Msr1*	Sense	5’-TCCCTTCCTCACAGCACTAA-3	60
	Antisense	5’-GGAAGCGTCCCGTGTCTAT-3	
*Abca1*	Sense	5’-AAAACCGCAGACATCCTTCAG-3	60
	Antisense	5’-CATACCGAAACTCGTTCACCC-3	
*Abcg1*	Sense	5’-GGTCCTGACACATCTGCGAA-3	60
	Antisense	5’-CAGGACCTTCTTGGCTTCGT-3	
*Scarb1*	Sense	5’-GGCTGTGGGAACTCTAGCTG-3	60
	Antisense	5’-CCGTCCTCTGTGGGAACTAA-3	
*Gapdh*	Sense	5’- ACTTTGTCAAGCTCATTTCC-3’	60
	Antisense	5’- TGCAGCGAACTTTATTGATG-3’	

### Oil red O staining

Aortas were opened longitudinally from the heart to the iliac arteries and fixed with 10% formalin, and lesions were stained with Oil-red O for en face analysis as previously described^54^. Optimal cutting temperature (OCT) compound-embedded hearts were sectioned and stained with Oil red O, and atherosclerotic lesions were quantified as previously described^55–58^. The lesions were assessed and quantified blindly by two independent observers.

### Histopathology analysis

Mouse aorta samples were isolated, fixed with 10% formaldehyde in PBS at room temperature for 24 h, embedded in paraffin, and sectioned at 7 μm. Aorta morphological histomorphometric characters were analyzed via hematoxylin/eosin (H&E) staining. Picrosirius red stain kit (Polysciences Inc. Warrington, PA, USA) was performed to identify collagen fibers, following the manufacturer’s instructions. H&E staining and Picrosirius red-stained collagen were observed under light microscopy (Leica Imaging Systems, Cambridge, UK). The digital photomicrographs were quantified with Image-Pro Plus Software (Media Cybernetics, USA). The stainings were assessed and quantified blindly by two independent observers.

### Atherosclerotic plaque morphology histomorphometric analysis

Atherosclerotic plaques at the aortic root were sectioned as previously described^57,59^. Plaque morphological histomorphometric characters were analyzed by hematoxylin and eosin (H&E) staining^57^. Plaque composition of lipid-rich cores, collagen, smooth muscle cell, and macrophage contents was analyzed by Oil red O staining, trichrome staining, immunofluorescence staining for α-SMA and CD68, respectively. The stainings were assessed and quantified blindly by two independent observers. Plaques stability was evaluated by comparing the ratios of the plaque components mentioned above to the entire plaques. The histological vulnerability index was also calculated as previously described^60^, following the formula: (vulnerability index) = (macrophage area + lipid area)/(smooth muscle cell area + collagen area).

### Immunostaining

Immunohistochemical staining was performed on 7-µm sections of aortas embedded in paraffin. Rehydrated antigen-retrieved sections were incubated with antibodies against CD68 (1:100 dilution) and visualized by the avidin–biotin complex method using the chromogen diaminobenzidine (Zhongshan Golden Bridge Biotechnology, Beijing, China).

Immunofluorescence staining of atherosclerotic lesions was performed on 10-µm sections of heart roots freshly embedded in OCT, as described previously^54,59^. Sections were incubated with antibodies against IMD (1:50 dilution), CD68 (1:100 dilution), α-SMA (1:100 dilution), GRP78 (1:200 dilution), ATF4 (1:100 dilution), cleaved ATF6 (1:100 dilution), CHOP (1:50 dilution), cleaved caspase-3 (1:100 dilution), ASC (1:100 dilution), IL-1β (1:100 dilution), and IRE1α (1:100 dilution) at 4 °C overnight, and were then rinsed with PBS and incubated with fluorescein-labeled secondary antibodies (EarthOx Life Science, Millbrae, CA, USA). The nuclei were stained by mounting the slides with Hoechst (Solarbio Science & Technology Co., Ltd, Beijing, China). Images were acquired with a Leica fluorescence microscopy (Leica Imaging Systems, Cambridge, UK).

Negative controls were omission of the primary antibody with secondary antibody only; in all cases, negative controls showed insignificant staining. Immunostaining data were quantified with blinding to the treatment group.

### TdT-mediated dUTP nick-end labeling (TUNEL) staining and Hoechst staining

The apoptotic cells in the mouse aortas were identified by TUNEL staining according to a kit (Roche Applied Science, Indianapolis, USA). Frozen aortic sections were fixed at 4% paraformaldehyde for 20 min. After being washed for 30 min with PBS, sections were incubated in permeabilization solution (0.1% TritonX-100, 0.1% sodium citrate, freshly prepared) for 2 min, then TUNEL reaction mixture was added (mix enzyme solution with label solution) and sections were incubated in a dark humidified chamber at 37 ^o^C for 60 min. Sections were washed with PBS and photographed under a fluorescence inverted microscope (Leica Imaging Systems, Cambridge, UK). Proteolytic activity was detected as bright green fluorescence (490 nm).

Hoechst staining of macrophages involved a Hoechst staining kit (Beyotime Institute of Biotechnology). Fixed cells were washed with PBS and stained with Hoechst (5 mg/mL) for 5 min. Cells were washed twice with PBS and were then observed under a fluorescence microscope for condensed or fragmented apoptotic nuclei. The stainings were assessed and quantified blindly by two independent observers.

### Enzyme-linked immunosorbent assay (ELISA)

Plasma IL-1β was measured by the commercial ELISA kit from Boster Biological Technology, Co. (Wuhan, China) as previously described^61^.

### Statistical analysis

All data are expressed as mean ± SD. Statistical analysis involved unpaired two-tailed Student’s *t* test for comparison of two groups when data normally distributed, the Mann–Whitney test for comparison of two groups when data not normally distributed, or one-way ANOVA for multiple groups, with post-hoc analysis by Student–Newman–Keuls test. *P* < 0.05 was considered statistically significant. *N* numbers of each group are listed in figure legends. All the statistics were analyzed using GraphPad Prism version 7.0 (GraphPad Software Inc., San Diego, CA, USA).

## Supplementary information

Supplementary figure legends

Supplementary Figure 1

Supplementary Figure 2

Supplementary Figure 3

Supplementary Figure 4

Supplementary Figure 5
