# Supplementary material for: Intermedin1-53 attenuates atherosclerotic plaque vulnerability by inhibiting CHOP-mediated apoptosis and inflammasome in macrophages
Source: Cell Death Dis. 2021 May 1;12(5):436. doi: 10.1038/s41419-021-03712-w (PMC8088440; doi:10.1038/s41419-021-03712-w)
Supplement: Supplementary file 1 — Supplementary figure legends [file 41419_2021_3712_MOESM1_ESM.docx]

Supplementary Figure Legends for

**Intermedin_1-53_ attenuates atherosclerotic plaque vulnerability by inhibiting CHOP-mediated apoptosis and inflammasome in macrophages**

Jin-Ling Ren, Yao Chen, Lin-Shuang Zhang, Ya-Rong Zhang, Shi-Meng Liu, Yan-Rong Yu, Mo-Zhi Jia, Chao-Shu Tang, Yong-Fen Qi, Wei-Wei Lu

**Supplementary figure 1.**

**IMD_1-53_ inhibited the development of atherosclerosis.** Eight-week-old male ApoE^-/-^ were fed a standard chow diet (con) or a high-fat diet (HFD) for 16 weeks. After 10 weeks of HFD feeding, ApoE^–/–^ mice received either PBS or intermedin_1-53_ (IMD_1-53_) during the left 6 weeks of high-fat diet feeding. (A) Representative images and quantification data of intermedin (IMD) immunohistochemical staining at aortic root of mice from con and HF. Black arrows indicate IMD-positive area. Scale bars, 200 μm. (B) Quantitative real-time PCR of IMD mRNA expression. n=6. Data are mean ± SD. ^**^*P* < 0.01 compared with con; Student’s *t*-test. (C) Body weight of mice from each group. (D) Plasma total cholesterol (TC) and triglyceride (TG) levels were measured. Lipoprotein fractions (LDL and HDL) were isolated and the cholesterol levels of each fraction were measured. n=8~12. Data are mean ± SD. ^**^*P* < 0.01, ^*^*P* < 0.05; one-way ANOVA.

**Supplementary figure 2.**

**Endoplasmic reticulum stress (ERS) is markedly increased in advanced atherosclerotic plaque.** Eight-week-old male ApoE^-/-^ were fed a standard chow diet (con) or a high-fat diet (HFD) for 16 weeks. After 10 weeks of HFD feeding, ApoE^–/–^ mice received either PBS or intermedin_1-53_ (IMD_1-53_) during the left 6 weeks of high-fat diet feeding. (A) Representative images of ATF4 and CD68 immunofluorescence staining showing lesion ATF4+ area in green and CD68+ macrophage area in red, and their merging images. (B) Western blot analysis of protein expression of GRP78, ATF6 and ATF4 in macrophages treated with ox-LDL of different concentrations. β-actin was a control for protein loading. Results are representative of 3 experiments. Densitometric analysis of protein levels is shown as ratio to β-actin. Data are mean ± SD. ^*^*P* < 0.05, ^**^*P* < 0.01, ^***^*P* < 0.001 compared with 0 ug/mL; one-way ANOVA. (C) Western blot analysis of protein expression of GRP78 and ATF6 in macrophages treated with ox-LDL for different times. β-actin was a control for protein loading. Results are representative of 4 experiments. Densitometric analysis of protein levels is shown as ratio to β-actin. n=4. Data are mean ± SD. ^*^*P* < 0.05, ^**^*P* < 0.01 compared with 0 h; one-way ANOVA.

**Supplementary figure 3.**

**Inhibition of endoplasmic reticulum stress (ERS) reduced atherosclerotic lesions formation at aortic root of ApoE^-/-^ mice.** Eight-week-old male ApoE^-/-^ were fed a standard chow diet (con) or a high-fat diet (HFD) for 16 weeks. After 10 weeks of HFD feeding ApoE^–/–^ mice received either PBS or Taurine (Tau) during the left 6 weeks of high-fat diet feeding. (A) Gross view of en face-prepared aortas stained with Oil Red O. The red dots are lesions stained positively for Oil Red O. Yellow arrows pointed to the Oil red O-stained lesions. (B) Representative image of Oil red O-stained aortic root lesion. Scale bars, 500 μm. (C) Quantification of Oil red O positive area. (D) Representative image of H&E stained aortic root lesion. Black arrows indicate the atherosclerotic lesions. Scale bars, 500μm. (E) Quantification of aortic root lesion area. n=7~10. Data are mean ± SD. ^*^*P* < 0.05, ^**^*P* < 0.01; one-way ANOVA.

**Supplementary figure 4.**

**Inhibition of endoplasmic reticulum stress (ERS) attenuated atherosclerotic plaque vulnerability at aortic root of ApoE**^-^**^/-^ mice.** Eight-week-old male ApoE^-/-^ were fed a standard chow diet (con) or a high-fat diet (HFD) for 16 weeks. After 10 weeks of HFD feeding ApoE^–/–^ mice received either PBS or Taurine (Tau) during the left 6 weeks of high-fat diet feeding. (A) Necrotic core area at aortic root were measured. Representative H&E-stained sections from each group are displayed next to the quantification data. Black arrows indicate the necrotic core. Scale bars, 200 μm. (B) Collagen area at aortic root were measured. Representative picrosirius red-stained sections from each group are displayed next to the quantification data. Scale bars, 200 μm. n=9~10. Data are mean ± SD. ^*^*P* < 0.05, ^**^*P* < 0.01; one-way ANOVA. (C-D) Representative images of CD68 (C) and α-SMA (D) immunohistochemical staining (left) and quantitative analysis of macrophage and vascular smooth muscle cell (VSMC) contents (right) at aortic root of mice from each group. Black arrows indicate cells stained positively for CD68 or α-SMA. Scale bars, 200 μm. (E) Quantitative analysis of plaque vulnerability index at aortic root of mice from each group. n=6. Data are mean ± SD. ^*^*P* < 0.05, ^**^*P* < 0.01 compared with con, ^#^*P* < 0.05, ^##^*P* < 0.01 compared with HF group; one-way ANOVA.

**Supplementary figure 5.**

**CHOP was upregulated in macrophages of advanced lesions in ApoE^-/-^ mice.** (A) Genotyping results of ApoE^-/-^CHOP^-/-^ mice. (B) Representative images of CHOP and CD68 immunofluorescence staining showing lesion CHOP+ area in green and CD68+ macrophage area in red, and their merging images. (C) Representative images of cleaved caspase-3 and CD68 immunofluorescence staining showing lesion cleaved caspase-3+ area in green and CD68+ macrophage area in red, and their merging images. (D) Western blot analysis of protein expression of CHOP in macrophages treated with ox-LDL of different concentrations. β-actin was a control for protein loading. Results are representative of 4 experiments. Densitometric analysis of protein levels is shown as ratio to β-actin. n=4. Data are mean ± SD. ^**^*P* < 0.01 compared with 0 μg/mL; one-way ANOVA. (E) Western blot analysis of protein expression of CHOP in macrophages treated with ox-LDL for different times. β-actin was a control for protein loading. Results are representative of 4 experiments. Densitometric analysis of protein levels is shown as ratio to β-actin. n=4. Data are mean ± SD. **P* < 0.05 compared with 0 h; one-way ANOVA.
