# Supplementary figures and images for: Intermedin1-53 attenuates atherosclerotic plaque vulnerability by inhibiting CHOP-mediated apoptosis and inflammasome in macrophages
Source: Cell Death Dis. 2021 May 1;12(5):436. doi: 10.1038/s41419-021-03712-w (PMC8088440; doi:10.1038/s41419-021-03712-w)

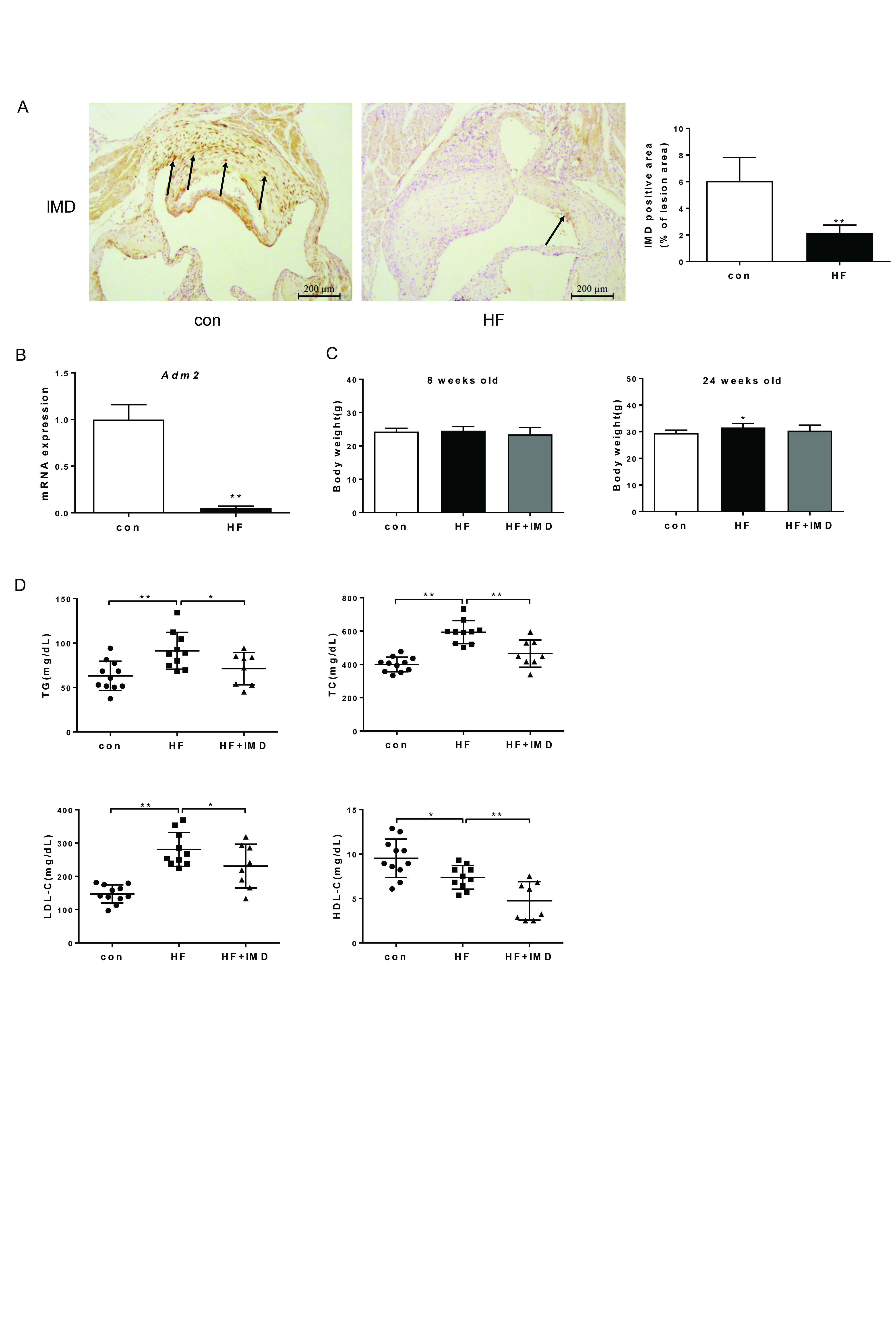

Supplement: Supplementary file 2 — Supplementary Figure 1 [file 41419_2021_3712_MOESM2_ESM.tif]

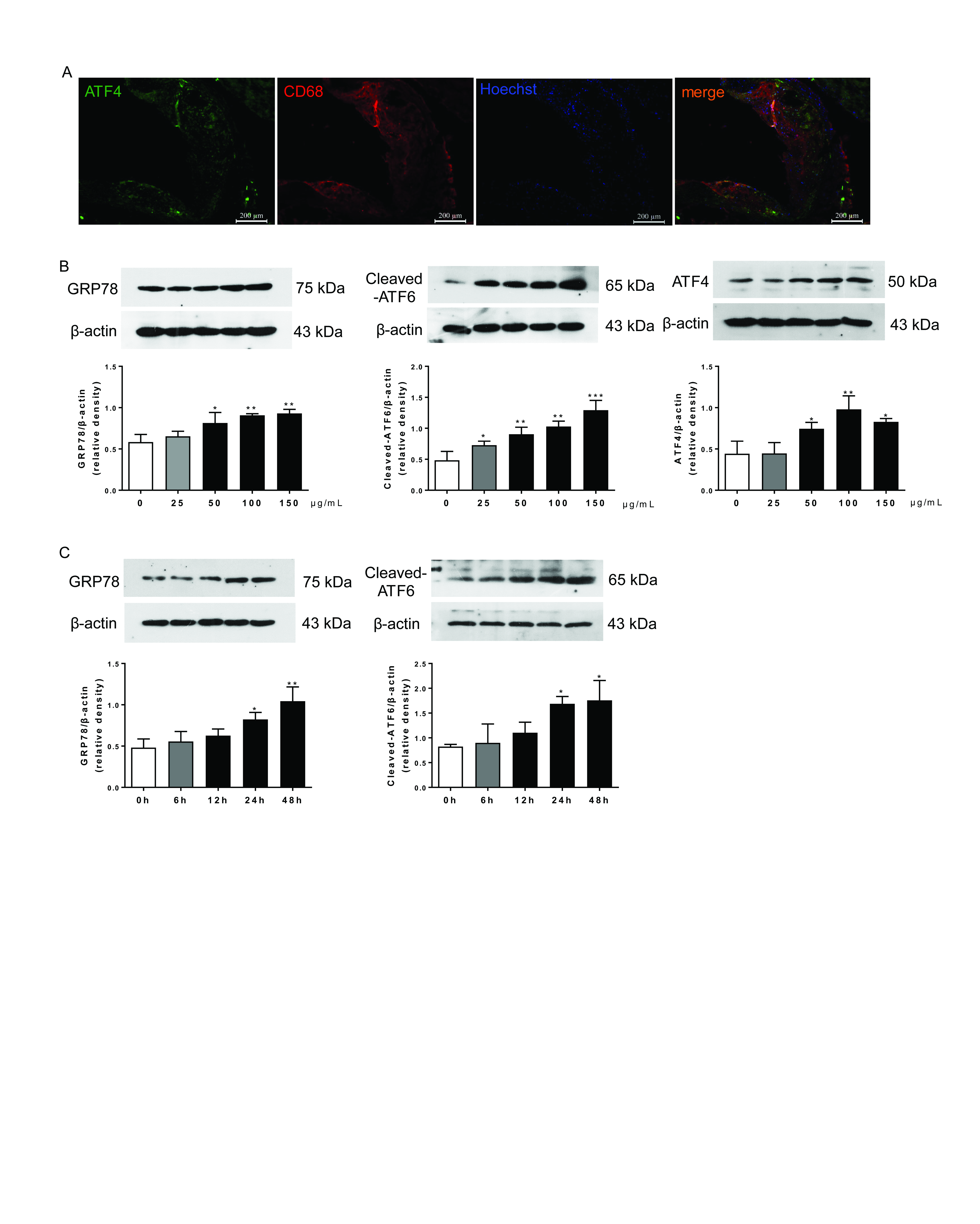

Supplement: Supplementary file 3 — Supplementary Figure 2 [file 41419_2021_3712_MOESM3_ESM.tif]

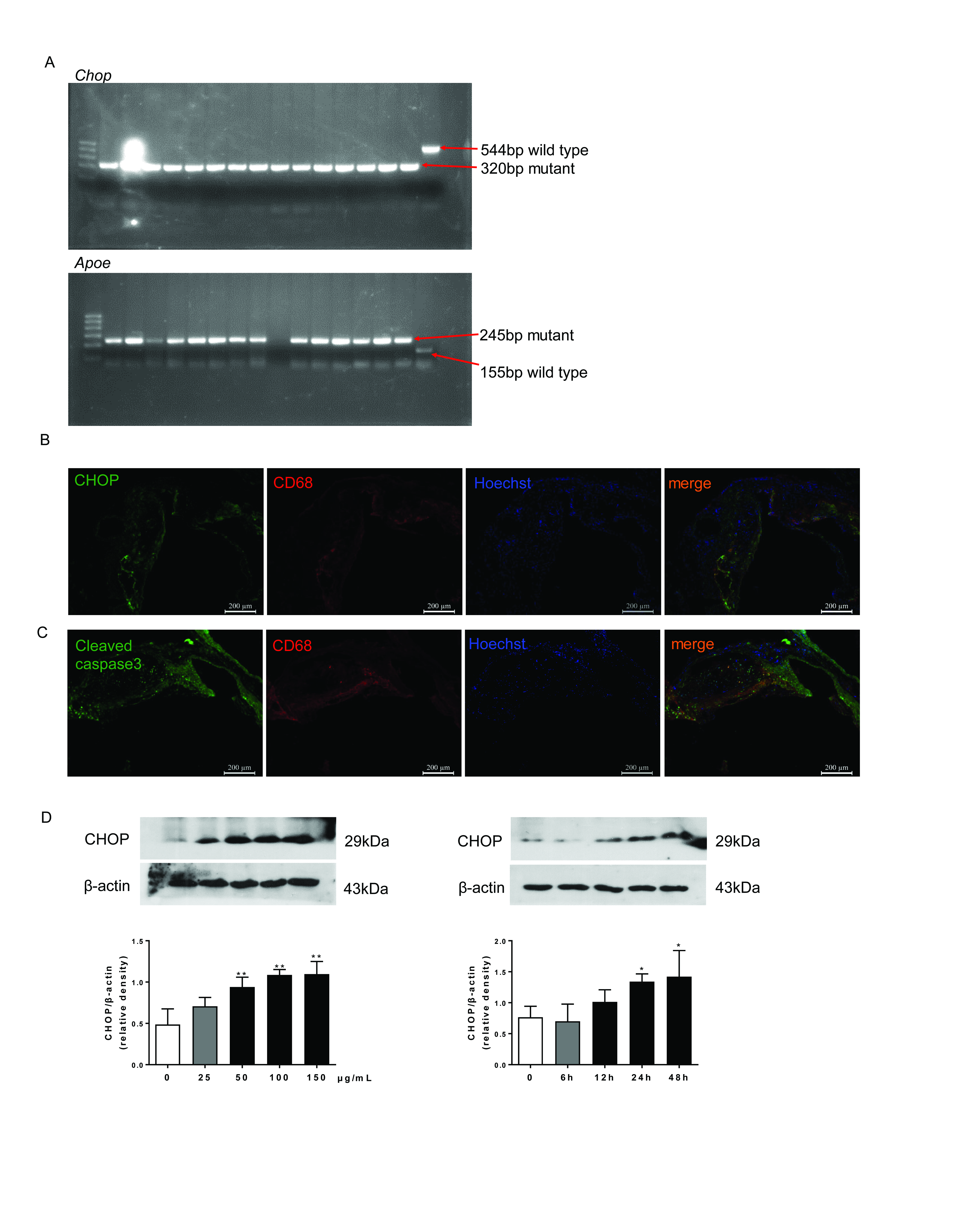

Supplement: Supplementary file 6 — Supplementary Figure 5 [file 41419_2021_3712_MOESM6_ESM.tif]
